# Echinoderm conundrums: Hox genes, heterochrony, and an excess of mouths

**DOI:** 10.1186/2041-9139-5-46

**Published:** 2014-12-22

**Authors:** Thurston Lacalli

**Affiliations:** Biology Department, University of Victoria, Victoria, BC V8W-3N5 Canada

**Keywords:** Body plan, Echinoderms, Heterochrony, *Hox* genes, Hydrocoel

## Abstract

Two issues relating to the translocation of anterior *Hox* genes in echinoderms to the 5’ end of the Hox cluster are discussed: i) that developmental changes associated with fixation to the substratum have led to an acceleration of mesodermal development relative to that of ectoderm, resulting in a mismatch of anteroposterior registry between the two tissues and a larger role for mesoderm in patterning control, and ii) whether this helps explain the ability of some echinoderms to form separate mouths at different locations, one for the larva and one for the juvenile rudiment. Freeing the mesoderm from ectodermal influences may have encouraged morphogenetic innovation, paralleling the situation in tunicates, where an early genomic (or genomic and developmental) change has allowed the body to evolve in novel ways.

## Background

This essay began as a brief Comment on an informative review of echinoderm *Hox* genes by David & Mooi recently published in this journal [1], but then morphed into a somewhat broader treatment of echinoderm development. Echinoderms are exceptional for the degree to which they have diverged from what we suppose to have been the ancestral bilaterian body plan. David & Mooi argue this divergence correlates with translocation of the anterior *Hox* genes to the 5’ end of the cluster, along with their inversion, so as to take them completely out of the game when it comes to early patterning events. To go beyond the fact of such a change to examine more fully how and why it may have occurred (a strategy outlined by Jenner [2], among others), a number of related issues need to be addressed. Two such issues concern me here, my elephants in the room, so to speak, since, though clearly relevant, they are often left out of discussions of echinoderm origins and evolution. These are, i) the increased degree of mesodermal control over development in echinoderms relative to that of ectoderm, and ii) whether this relates to the ability of asteroids and echinoids to form two separate mouths in one individual (one for the larva and one for the developing juvenile), an example of how, among other curiosities, echinoderms seem to violate the usual rules of body patterning.

### Heterochrony and its consequences

My first elephant is the restriction of early Hox expression to mesodermal structures, namely coeloms, in patterns that conserve the ancestral linear anteroposterior (A/P) sequence within the mesoderm. Since the *Hox* genes that are expressed are trunk-specific, the situation can be interpreted as evidence that, though the trunk has been lost as an identifiable body part, some of its constituent mesoderm has been retained and co-opted to enclose the visceral organs and to support the stalk as it evolved. Ectodermal Hox expression is delayed in time, restricted to the developing oral domain, and follows a radial rather than linear plan [3, 4]. This suggests that ectodermal Hox expression has either simply been switched off during early development, or is delayed, perhaps in part due to translocation. Regardless of mechanism, the result is a form of heterochrony in which mesodermal patterning and development is accelerated relative to that of ectoderm.

This contrasts dramatically with the situation in other deuterostome phyla, where early patterning events depend on Hox expression in ectoderm and neural tissue either exclusively or in combination with co-linear expression in mesoderm, as occurs in vertebrate somites. A rationale for seeing this as related to alterations in body plan is as follows: in all deuterostomes except echinoderms, the ectoderm and mesoderm show a high degree of A/P registry, that is, defining “anterior” from the developmental genetic perspective as being the site of the larval apical organ, a key landmark across phyla [5], the A/P identity of adjacent tissue layers is roughly in register along the whole length of the body, and development follows an anterior to posterior sequence. This registry is lost in echinoderms that undergo rotation of the coelomic and visceral organs (see [6] for a detailed account) which, if basal to echinoderms as David & Mooi argue, is the situation in all living echinoderms, and some if not all of the fossils (there are divergent views on this last point, see [7]).

**Figure 1:**
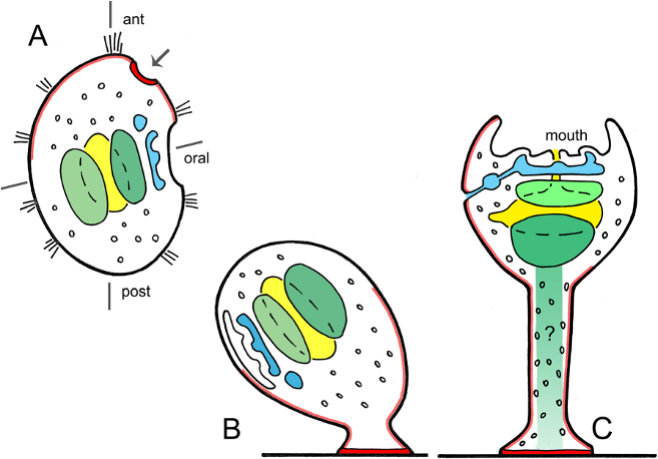
**Settlement and metamorphosis in the crinoid** ***Oxycomanthus japonicus***, **modified from [**[9]**]. (A)** A swimming, non-feeding doliolaria larva near settlement, with the A/P axis indicated. At this stage the vestibular depression that defines the future oral surface lies at a roughly 90° angle from this axis with the preoral adhesive organ (arrow, in red) located just above it. At settlement **(B)**, the adhesive organ attaches to the substratum and subsequently acts as the attachment disc, and the preoral region begins to narrow and elongate to form the stalk. The vestibule has closed over internalizing the oral surface and the future site of mouth formation. **(C)** Late cystidean stage, with the visceral organs (coeloms and gut) rotated so the vestibular sac, now open to expose the mouth, points upward. Colors indicate the developing gut (yellow), axocoel and hydrocoel (light blue, the former is the smaller), and the left and right somatocoels (green, the latter is darker). A lighter red is used to trace the expected extent of the preoral larval ectoderm during rotation and stalk elongation, assuming its expansion occurs without distortion or significant cell rearrangement, though precisely how early ectodermal domains map to later stages is not known. Nevertheless, it does seem that the ectoderm of the stalk comes largely from preoral larval ectoderm, whereas, in contrast, internal structures, including all or parts of the chambered organ, and possibly the ligaments, appear to derive from the right somatocoel, as indicated by the green rectangle. The query is a reminder that these are all suppositions based on the morphology that have yet to be verified experimentally.

The loss of registry is best seen in crinoids during fixation and early morphogenesis of the cystidean stage ([8–10] and Figure 1): the anterior portion of the larval body forms a good part of the ectoderm of the stalk, which in terms of A/P identity ought to be anterior and largely preoral, but this is then invaded by cells derived from the right somatocoel, whose Hox expression indicates a much more caudal origin, to form stalk structures, including at least parts of the chambered organ and possibly ligaments and muscles. The ectodermal component of the cystidean oral region, located at its distal end, and the basal adhesive disc, are therefore both considerably more anterior in terms of their A/P origin than the mesodermal derivatives they enclose.

With a decoupling of the ectoderm from the mesoderm in terms of spatial registry, there is potentially a problem if both continue to play an active role in patterning, as adjacent tissues representing different A/P levels could, in principle, generate conflicting signals. A way to avoid this is to have one tissue dominate, and for the linear phase of patterning in echinoderms it is evidently the mesoderm that has done so. This could explain why the anterior *Hox* genes have been translocated, as this would effectively suppress their expression, in ectoderm and elsewhere, by delaying it. The result is that mesodermal structures of caudal origin would begin their differentiation within an envelope of ectoderm that lacks any Hox-dependent A/P identity, which may mean the latter tissue remains in what is effectively, at least in this respect, a larval or embryonic state.

Giving mesoderm the active role in patterning is perhaps not surprising for a phylum in which the body depends on a support system of mesodermal origin. It also accords with the evidence for the organizing capacity of mesodermal structures (e.g., the coeloms) demonstrated in the classical regeneration experiments of Hörstadius [11], and the key role the hydrocoel plays in the induction of oral structures. It would clearly be useful to revisit such experiments using more modern tools to show, through the resulting expression patterns, the more immediate consequences of removing or relocating selected structures like the hydrocoel, or of over- and under-expressing upstream control genes, including *Hox* genes.

### Duplicating the mouth

This brings me to the second elephant, perhaps most evident to those, like myself, concerned with chordate origins and, specifically, the repositioning (or re-evolution in some scenarios) of the mouth following the dorsoventral inversion that is thought to have occurred at the base of the chordate lineage [12]. The remarkable feature of echinoderms, from this perspective, is that they can form two separate mouths in one body. This occurs in the larvae of both echinoids and asteroids when the juvenile rudiment is formed, so that a second mouth develops under the control of the hydrocoel, but at some distance (though still within the oral field) from the mouth used by the larva to feed. The ectoderm evidently plays some role in this process, since a vestibule will form at the normal position in echinoid larvae even if the hydrocoel is absent, but without a hydrocoel, the vestibule fails to develop further [11]. The endoderm also probably has a role in forming the mouth opening, as some endodermal tissue remains closely associated with the hydrocoel ([13] and VB Morris, unpublished data). Regardless of details, the freedom to produce mouths at novel sites seems best explained if the role of ectoderm in patterning is reduced so that both mouth formation and the differentiation of associated oral structures are controlled by internal tissues, whether mesoderm, endoderm, or a combination of the two. A mouth would then be formed wherever these latter tissues generate appropriate signals, and a duplicate mouth could then evolve by having such signals re-directed to a new location.

This situation presents both a problem and an opportunity. The problem is one of homology, i.e., of comparing body plans between echinoderms and other phyla when it is not clear which mouth is the relevant one for comparison. When there are two mouths in one animal, as in echinoid development, is each a homolog of the other, and should only one (if so, which one) or both be considered the true homologs of, say, the chordate mouth, whose homology with mouths in other phyla is in any case a matter of some uncertainty [14]. The opportunity comes from the involvement of similar genes in symmetry control and mouth formation across phyla, notably *nodal*. Nodal signaling is crucial to developmental asymmetry in both echinoids and amphioxus [15, 16], and differences between these taxa in the response of the system to perturbation can be used to reveal something of its evolution. Since echinoderms present a range of variants, depending on class, of either a single mouth or separate mouths for the larva and juvenile, positioned on the bilateral axis or off it, there is considerable scope for investigating the precise role *nodal* plays in altering symmetry in echinoderms and, directly or indirectly, the location of the mouth, whether single or plural.

Some past ideas on the ability to produce extra mouths (e.g., [17]) have speculated that it might reflect an evolutionary past that included asexual propagation as a means of reproduction. The case made by David & Mooi suggests an alternative that reverses the polarity of this scenario, so that asexual propagation (which occurs in some echinoderm larvae, see [18]) would be a consequence of a deeper developmental genetic change that allowed for subsequent alteration of the way mouth position is determined. This is comparable in some ways to the fundamental developmental and genomic change postulated to have freed tunicates to evolve a whole set of body plan innovations [19]. In tunicates, it seems to be the compression in time and lineage-dependence of development that made the slower unfolding of the ancestral patterning program unnecessary, hence leading to loss or alterations in the genes responsible, including members of the Hox cluster. The crucial event in echinoderms would seem instead to relate, either as a consequence or correlate, to a loss of A/P registry, which then led to a more direct control by internal tissues over morphogenetic events, including mouth formation. From the perspective of an amphioxus biologist, where both the genome and morphology are comparatively conservative, the ability of echinoderms to remake their morphology with such apparent ease is remarkable.

## Conclusions

There are distinctive features of the echinoderm Hox cluster and of Hox expression that correlate with the loss of axial registry between embryonic ectoderm and mesoderm that would necessarily have accompanied the evolution of crinoid-type settlement and organ rotation. A case can be made that the mesoderm may, at that time, have taken on a greater role in morphogenetic control, which would explain the restriction of early Hox expression to mesoderm, as well as the control that the latter exercises over morphogenesis as demonstrated by experiment. For mouth formation, increased mesodermal control may provide an explanation for how two separate mouths can be formed in the same animal if the hydrocoel has thereby been freed of constraints that would have prevented it from inducing a second mouth. It is not clear whether the ability to produce extra mouths is something that other bilaterian lineages could readily have evolved given sufficient selective advantage, or whether it has arisen in the case of echinoderms only because of a very specific, unique set of evolutionary circumstances.

## Abbreviations

A/P: Anteroposterior.
